# The functional convergence of antibiotic resistance in β‐lactamases is not conferred by a simple convergent substitution of amino acid

**DOI:** 10.1111/eva.12835

**Published:** 2019-07-18

**Authors:** Vivek Keshri, Kevin Arbuckle, Olivier Chabrol, Jean‐Marc Rolain, Didier Raoult, Pierre Pontarotti

**Affiliations:** ^1^ Aix‐Marseille Université, IRD, APHM, Microbe, Evolution, PHylogenie, Infection, IHU ‐Méditerranée Infection Marseille France; ^2^ Department of Evolution, Ecology and Behaviour University of Liverpool Liverpool UK; ^3^ Department of Biosciences, College of Science Swansea University Swansea UK; ^4^ Aix‐Marseille Université, I2M, UMR‐CNRS 7373, Evolution Biologique et Modélisation Marseille France; ^5^ CNRS Marseille France

**Keywords:** antibiotic resistance, convergent evolution, β‐lactam antibiotics, β‐lactamase

## Abstract

Bacterial resistance to antibiotics is a serious medical and public health concern worldwide. Such resistance is conferred by a variety of mechanisms, but the extensive variability in levels of resistance across bacteria is a common finding. Understanding the underlying evolutionary processes governing this functional variation in antibiotic resistance is important as it may allow the development of appropriate strategies to improve treatment options for bacterial infections. The main objective of this study was to examine the functional evolution of β‐lactamases, a common mechanism of enzymatic resistance that inactivates a widely used class of antibiotics. We first obtained β‐lactamase protein sequences and minimal inhibitory concentration (MIC), a measure of antibiotic function, from previously published literature. We then used a molecular phylogenetic framework to examine the evolution of β‐lactamase functional activity. We found that the functional activity of antibiotic resistance mediated by β‐lactamase has evolved in a convergent manner within molecular classes, but is not associated with any single amino acid substitution. This suggests that the dynamics of convergent evolution in this system can vary between the functional and molecular (sequence) levels. Such disassociation may hamper bioinformatic approaches to antibiotic resistance determination and underscore the need for (less efficient but more effective) activity assays as an essential step in evaluating resistance in a given case.

## 1 INTRODUCTION

Antibiotics are medicines that fight bacterial infection, but there is a growing problem of antibiotic resistance when bacteria become able to resist the effects of antibiotics. Antibiotic‐resistant genes spread in the environment quickly due to overuse of antibiotics which imposes a strong selection pressure favoring resistance. For instance, humans use a range of antibiotics in agriculture and fish farming, as standard prophylactic measures, which leads to the widespread evolution of antibiotic‐resistant genes (Economou & Gousia, 2015). A common enzymatic resistance mechanism used by several groups of bacteria is the production of “β‐lactamase” enzymes that inactivate β‐lactam antibiotics (an important class of antibiotics which includes penicillins, cephalosporins, carbapenems, and monobactam). There are more than 1,300 natural lactamases, classified into four molecular classes (A, B, C, and D) (Ambler, 1980; Ambler et al., 1991; Bush, 2013; Bush & Jacoby, 2010; Bush, Jacoby, & Medeiros, 1995). The active site of class A, C, and D enzymes contains serine amino acid, while class B contains histidine along with Zn metal (Bebrone, 2007). In addition, class B (metallo‐β‐lactamase) has been classified into three subclasses B1, B2, and B3 (Bebrone, 2007). Based on substrate profile inhibitors, β‐lactamases are classified into functional subgroups such as 2a, 2b, 2be, and 2c. (Bush, 1989b, 1989a; Bush & Jacoby, 2010; Bush et al., 1995). Significant progress in sequencing technologies led to a large number of class A β‐lactamases in sequence database, in some cases being deposited under inappropriate classification (Philippon, Slama, Dény, & Labia, 2016). The class A enzymes can be further classified in subclasses A1 and A2 based on phylogenetic clustering (Philippon et al., 2016). Functional class B β‐lactamases, which appeared more than two billion years ago, suggest that resistance to antibiotics long predates their use in medicine (D’Costa et al., 2011). The functional classification of β‐lactamase and its correlation with their distinct molecular structure has been described by Bush et al. (1995), and the functional evolution of superfamily class B β‐lactamase has been studied by Alderson et al (Alderson, Barker, & Mitchell, 2014). However, Alderson et al. (Alderson et al., 2014) were unable to unambiguously demonstrate whether functional class B enzymes have one or two separate evolutionary origins (Alderson et al., 2014). Antibiotic resistance conferred by β‐lactamases spreads rapidly, (sometimes facilitated by horizontal gene transfer (Barlow,^2009^; Varsha K Vaidya, 2011; Doi, Adams‐Haduch, Peleg, & D'Agata, 2012; Warnes, Highmore, & Keevil, 2012). This information helps to understand the evolution of this resistance and to develop strategies for managing bacterial infections in humans, livestock, or other animals before they become a major epidemiological event. The number of β‐lactamase enzymes is regularly increasing, but their functional evolution remains unclear.

Convergent evolution refers to similarities that have evolved independently rather than being inherited from a common ancestor. “Convergence” is often used in a very general sense, but can apply to several types of characteristics, or levels, such as function and structure (Doolittle, 1994; Speed & Arbuckle, 2016). Several enzymes have developed the ability to catalyze the same reactions on separate occasions, such as those involved in the hydrolysis of peptide bonds (Rawlings & Barrett, 1993). Consequently, the structure of a protein's active site(s) determines the biochemical functions, but enzymes within the same structural class may have different roles or variable activity levels. Conversely, the convergence of enzymatic functions is a common phenomenon. For instance, the hydrolysis of peptide bonds has evolved many times and serine proteases have evolved at least three times (Doolittle, 1994).

The terms “iso‐convergent” and “allo‐convergent” can be applied to the study of convergent evolution to gain a finer‐scale understanding of the dynamics of convergence (Pontarotti & Hue, 2016). Iso‐convergent traits have converged from the same ancestral state (traditionally “parallel evolution”), whereas allo‐convergent traits have converged from different ancestral states. The allo‐convergent evolution of enzyme function results in two distinct, but sometimes joint, effects. For example, (a) nonhomologous enzymes deliver the same transformation, as expressed by the same four‐digit enzyme commission (EC) number; (b) the same (four‐digit EC) or related (same three‐digit EC) enzyme transformation is effected by a similar disposition of residues in the active site. These two situations are not exclusive because two enzymes are assigned to both classes if they perform exactly the same overall reaction with the same mechanism (Doolittle, 1994; Gherardini, Wass, Helmer‐Citterich, & Sternberg, 2007). The independent evolution of the same function, most often using different mechanisms, but sometimes using different catalytic mechanisms for essentially the same mechanism, is well documented for proteins of different families of nonhomologous enzymes (Gherardini et al., 2007). However, this phenomenon is rarer within homologous superfamilies. Iso‐convergence has been documented in the “HAD (haloacid dehalogenase) superfamily” of proteins (Burroughs, Allen, Dunaway‐Mariano, & Aravind, 2006). Burroughs et al. (2006) deduced the evolutionary history of the HAD superfamily and showed that it contains 33 large families of three kingdoms and that diversification within archaeal, bacterial, and eukaryotic domains originated from an ancestral common HAD superfamily.

This study investigated whether and to what extent convergent functional evolution occurs in β‐lactamases and whether this function can be predicted based on particular amino acid substitutions. We used the minimum inhibitory concentration (MIC) as a measure of functional activity of β‐lactamase enzymes, a standard technique in diagnostic laboratories to determine the susceptibility of organisms to antimicrobials (Andrews, 2001). The MIC is the lowest concentration of an antimicrobial that inhibits the visible growth of microorganisms after overnight incubation. Hence, high MICs correspond to a greater resistance to that antibiotic. A few studies have reported the presence of convergent evolution in β‐lactamases, but they have been based on the similarity of molecular sequences and the similarity of the structure of the active site of proteins rather than on demonstrated resistance to antibiotics, and have generally been small‐scale studies using a few enzymes (Hibbert‐rogers, Heritage, Todd, & Hawkey, 1994; 1997, & Perez‐Diaz, 1^1997^). Moreover, the phylogenetic relationships within the molecular classes of β‐lactamases remain poorly understood. Therefore, the current study investigates the functional evolution across the various superfamilies/classes of β‐lactamases for the first time.

## 2 MATERIALS AND METHODS

### 2.1 Data collection

The β‐lactamase amino acid sequences were retrieved from the Antibiotics Resistance Gene‐ANNOTation (ARG‐ANNOT) database (Gupta et al., 2014). A total of 1,147 sequences from the four molecular classes (A‐D) were recovered. All sequences were checked in the NCBI PubMed database (http://www.ncbi.nlm.nih.gov/pubmed/) to obtain the publication in which the sequence was described, from which we collected minimum inhibitory concentration (MIC) values of the respective β‐lactamase enzymes and β‐lactam antibiotics, whenever this information was available. Two types of MIC value were retrieved, with and without β‐lactamases, and we then calculated the fold change (MIC value of with β‐lactamase was divided by without β‐lactamase MIC) between them. A total of 218 sequences were selected with these MICs against different β‐lactam antibiotics. These MICs and fold change values are also available in the β‐lactamase database (Keshri et al., 2019). Further, the fold change MIC values were normalized on a log scale followed by convergent evolution study. The remaining sequences were excluded from the study because the sequences were published on the basis of homologous sequences. Some sequences were characterized on the basis of kinetic parameters, while others did not report MIC. Further information such as original published article, collected MIC values (with and without β‐lactamase), and other related information can be accessed in a β‐lactamase database (Keshri et al., 2019).

The MICs of the same species–strain, same experiment technique/conditions, and specific sufficient data are not available in the scientific community for a particular species–strain and experiment convergent evolution study. Therefore, it is not possible to analyze data in species and/or strain and expression system specific. Generally, for comparison of MICs between different experiments, it is also necessary to perform experiment in the same laboratory with similar experimental protocols for removing the technical variability. Unfortunately, this kind of specific datasets is not available. Because of these difficulties, we initiated to analyze all MIC data (regardless of specific species, strain, techniques, etc) of a particular class of β‐lactamase enzymes and correlate with their phylogenetic trees (each molecular class separately).

### 2.2 Multiple sequence alignment and phylogenetic inference

The multiple sequence alignment and phylogenetic tree reconstructions were conducted in MEGA6 version 6.05 (Tamura, Stecher, Peterson, Filipski, & Kumar, 2013). Sequences were aligned using the MUSCLE algorithm (Edgar, 2004) with default settings. The phylogenetic trees were constructed with the maximum‐likelihood method under a WAG + G+I amino acid substitution model (estimated to be the best substitution model by maximum‐likelihood‐based model comparison, also in MEGA6). Node support was estimated using 1,000 bootstrap replicates. The phylogenetic trees were midpoint‐rooted, and branch lengths of the phylogenetic trees were converted to relative time (“dated” with the total length of the tree equal to 1) using maximum penalized likelihood as implemented in the chronos function in the ape package of R 3.3.0 (Paradis, Claude, & Strimmer, 2004). In this article, we use the term “dated” for convenience (recognizing that no absolute date has been estimated). The chronos function allows different types of clock models including strict, correlated, relaxed clocks and discrete rate models. We fit each of these models, including discrete rate models with the number of rates ranging from a single rate for the tree (equivalent to a strict clock model) to a separate rate for each branch in the tree. In all cases, the relaxed clock model provided the best fit, as indicated by having the highest probability of penalized log‐likelihood, which was therefore used for the dating of all trees here.

### 2.3 Identifying convergent evolution

We estimated the ancestral functional activity over the tree using maximum‐likelihood (ML) estimation via the contMap function in the phytools package (Revell, 2012). Before conducting the analysis, we first logged the values of functional activity (MIC fold change), since the raw values varied exponentially, which can cause the estimation of ancestral states to perform poorly. Our results as presented are therefore based on estimated ancestral values of log (MIC fold change) rather than the raw activity values. A Python script (provided in Appendix S1) was used for the identification of amino acid residues that are potentially responsible for functional convergence. This script is able to detect signature amino acid residues among antibiotic‐resistant/susceptible enzymes.

## 3 RESULTS

### 3.1 Ancestral state estimation

Based on our ML estimation of ancestral enzyme activity, we observed convergent evolution of antibiotic resistance in each molecular class A‐D, which we discuss in details for each class below.

#### 3.1.1 Class A

**Figure 1:**
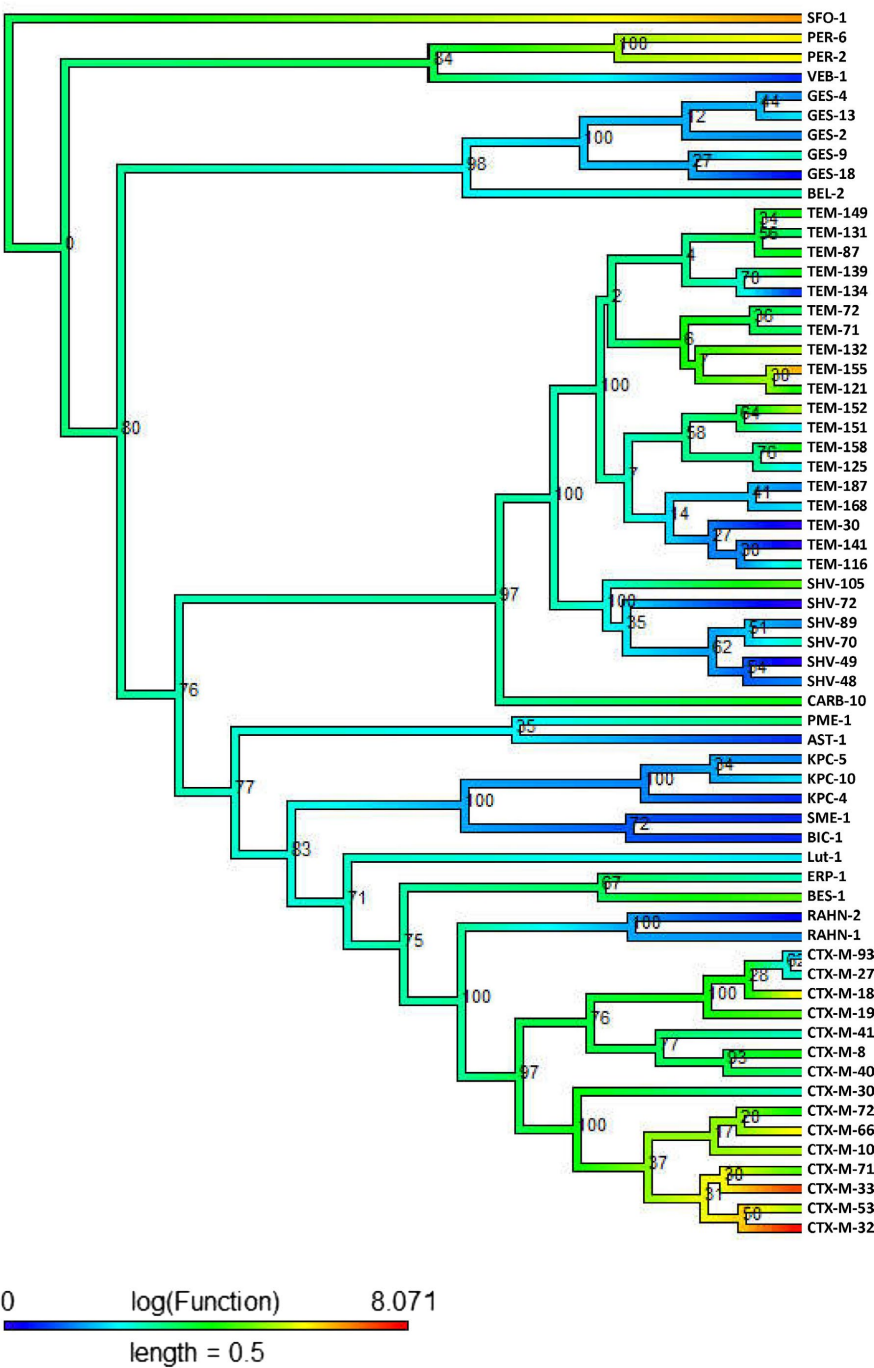
Class A (Cefepime): The midpoint‐rooted phylogenetic tree was constructed by maximum‐likelihood method based on the multiple sequence alignment. Bootstrap values are shown on each node. The phylogenetic tree contains class A β‐lactamases. The color of the branch (also in scale bar) indicates functional activity of enzymes against cefepime β‐lactam antibiotic. Blue through red color indicated susceptible to resistance functional activity

**Figure 2:**
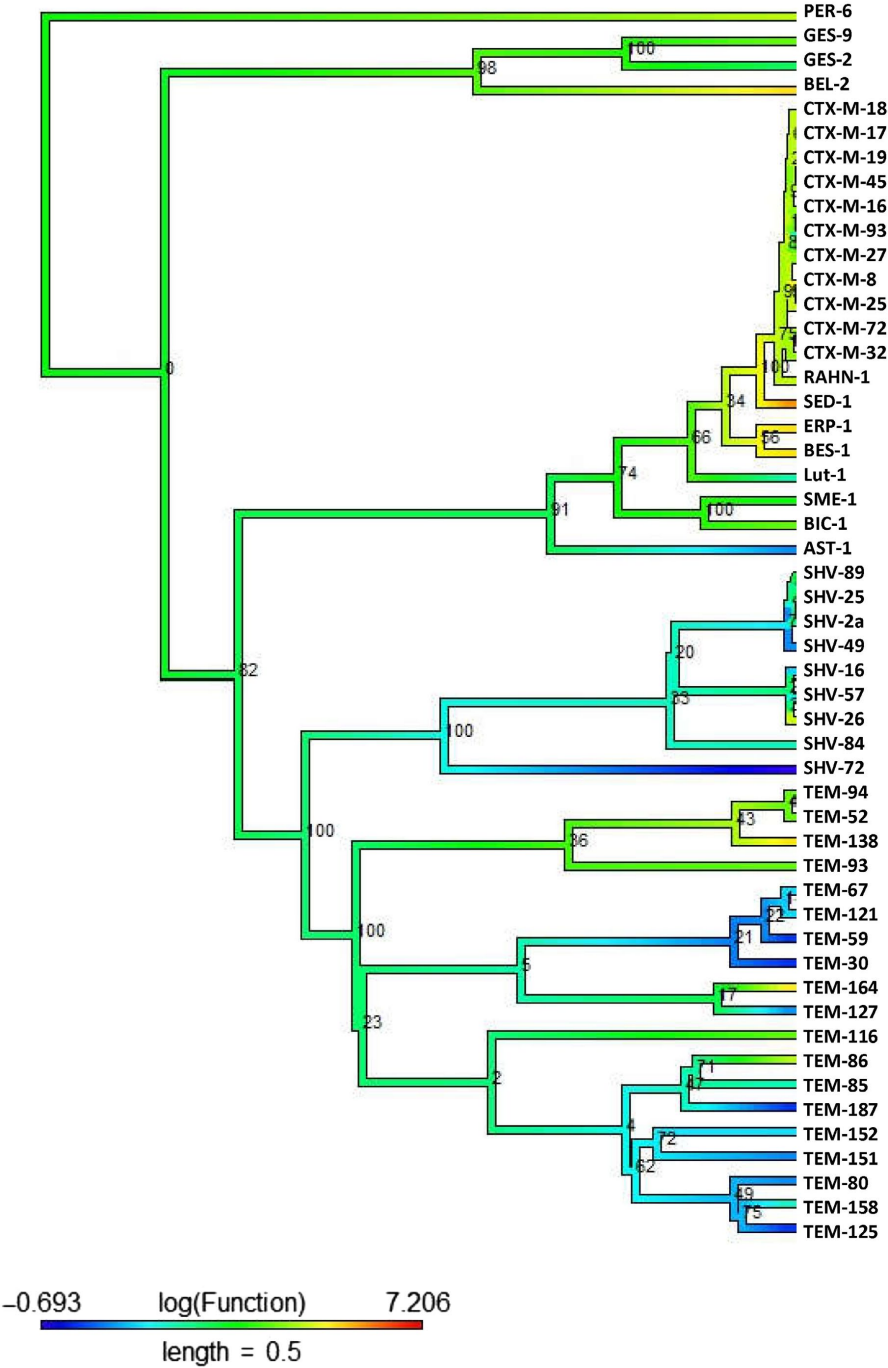
Class A (Cephalothin): The midpoint‐rooted phylogenetic tree was constructed by maximum‐likelihood method based on the multiple sequence alignment. Bootstrap values are shown on each node. The phylogenetic tree contains class A β‐lactamases. The color of the branch (also in scale bar) indicates functional activity of enzymes against cephalothin β‐lactam antibiotic. The color scheme and annotations are identical to those in Figure 1

The phylogenetic tree of class A β‐lactamases shows evidence for a convergent evolution of function with respect to the cefepime (Figure 1) and cephalothin antibiotics (Figure 2). The ancestral activity was estimated as moderately resistant (green color) to cefepime and cephalothin antibiotics. However, this may be partly due to the trend in the methods used to estimate ancestral states from continuous data to estimate near‐average values at the root, unless there is strong evidence to support otherwise. As they evolve, the function of certain enzymes (VEB‐1, GES‐2, GES‐18, TEM‐134, TEM‐30, TEM‐141, SHV‐72, SHV‐48, SHV‐49, AST‐1, KPC‐4, SME‐1, BIC‐1, and RAHN‐2 for cefepime, and TEM‐125, TEM‐187, TEM‐30, TEM‐59, and SHV‐72 for cephalothin) becomes susceptible to the antibiotic. On the other hand, the activity of enzymes CTX‐M‐19, CTX‐M‐8, CTX‐M‐40, CTX‐M‐72, TEM‐149, TEM‐131, TEM‐87, TEM‐71‐72, and CRAB‐10 for cefepime antibiotics remains unchanged and exhibits a similar functional affinity (moderately resistant to cefepime) when compared to their ancestors. Similarly, in the case of cephalothin, the CTX‐M, GES, PER group of enzymes has a similar scale of activity compared to that of their ancestor. Most of the modern enzymes reveal moderate resistance (green color) with respect to cephalothin (Figure 2). The CTX‐M‐32, CTX‐M‐33, and SFO‐1 indicate a strong resistance to cefepime antimicrobial agents, and this resistance appears to have evolved independently in each of those three enzymes (Figure 1). SED‐1 also has high resistance to cephalothin, and lower increases in resistance have evolved independently in additional lines.

#### 3.1.2 Class B

**Figure 3:**
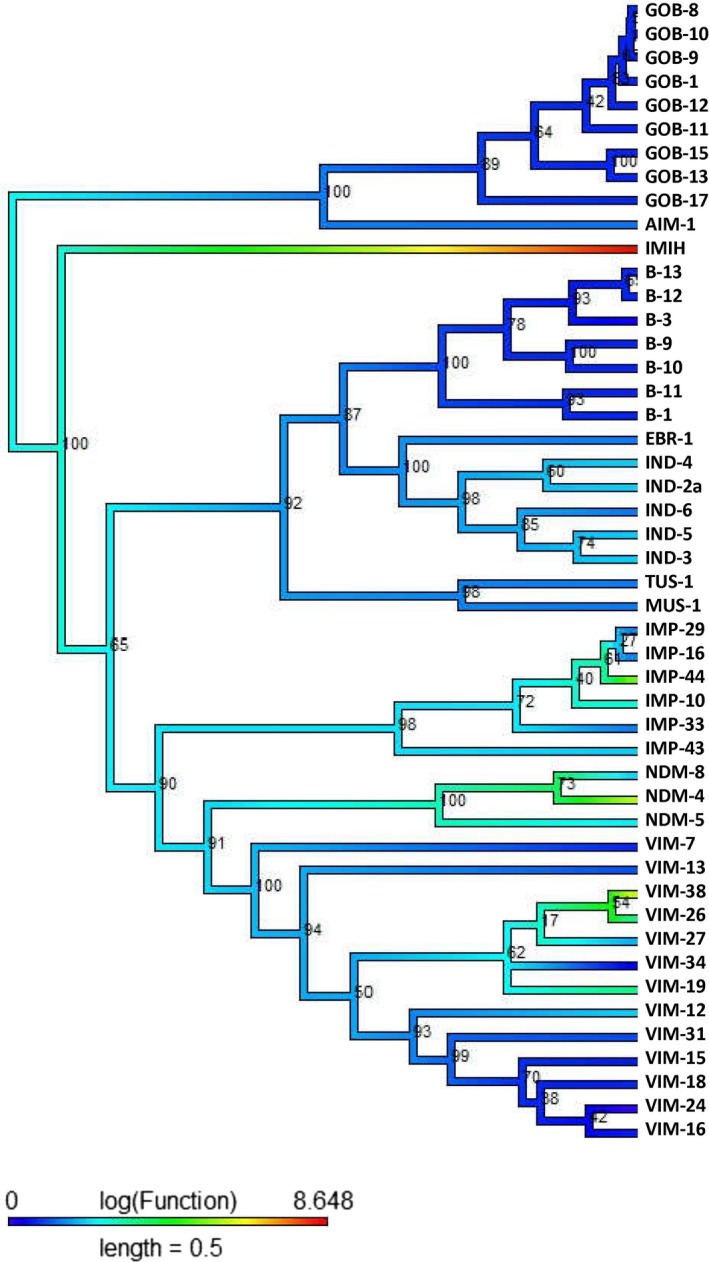
Class B (Piperacillin): The midpoint‐rooted phylogenetic tree was constructed by maximum‐likelihood method based on the alignment. Bootstrap values are shown on each node. The phylogenetic tree contains class B β‐lactamases. The color of the branch (also in scale bar) indicates functional activity of enzymes against piperacillin β‐lactam antibiotic. The color scheme and annotations are identical to those in Figure 1

Metallo‐β‐lactamase enzymes show evidence of a convergent evolution of both high levels of susceptibility and resistance to the antibiotic piperacillin (Figure 3). For instance, the GOB, B, TUS, MUS, and IMP groups (except IMP‐44), VIM‐7, VIM‐13, VIM‐34, VIM‐31, VIM‐15, VIM‐18, VIM‐24, and VIM‐16 show an increased susceptibility to piperacillin (decreasing functional affinity), while the NDM‐4, VIM‐38, VIM‐26, and IMP‐44 enzymes have independently evolved to be moderately resistant (green) to piperacillin. The IMIH enzyme appears to be highly resistant (red) to piperacillin.

The ancestral state shows susceptibility to imipenem antimicrobial agents (Figure S1), and this activity retained in some enzymes such as IMP‐10, IND‐3, VIM‐19, VIM‐38, and VIM‐26. Exceptionally, the functional affinities of group B (B‐1, B‐3, B‐9‐13) are entirely different from those of their last ancestors, and the enzyme reveals a further decrease in their functional (sensitive) affinity to imipenem. The IMIH β‐lactamase exhibits a strong resistance (strong functional affinity) to imipenem compared to ancestral. Meropenem also shows evidence of functional convergent evolution in β‐lactamase enzymes (Figure S2). The ancestral activity seems fairly susceptible to meropenem, while modern enzymes, such as GOB, B, VIM‐16, VIM‐24, VIM‐18, VIM‐15, VIM‐31, VIM‐13, and VIM‐7, are highly susceptible (blue color indicates lower MIC fold). However, it is more strongly supported by the fact that IMIH enzymes have a high resistance to meropenem, while VIM‐26, VIM‐38, IMP‐44, and NDM‐4 have a moderate resistance compared to IMIH. The above enzymes are distributed in different clades of the tree and display the independent evolution of the functions. The functional evolution of subclass B1 and B3 was estimated separately, and we observed that subclass B3 (Figures S7‐S9) does not exhibit any functional convergence, only a single origin of strong resistance in IMIH to imipenem and meropenem and in AIM‐1 to piperacillin. The subclass B1 exhibits a functional convergence in imipenem, meropenem, and piperacillin antibiotics (Figure S4‐S6). The evolution of the functional activity of resistance was estimated in the NDM and IMP enzymes compared to the antibiotics imipenem and meropenem. For piperacillin, the BlaB, MUS, and IMP enzymes showed a reduced activity as compared to the ancestral moderate resistance, while DIM, NDM‐4, and VIM showed a strong resistance which has convergently evolved.

#### 3.1.3 Class C

**Figure 4:**
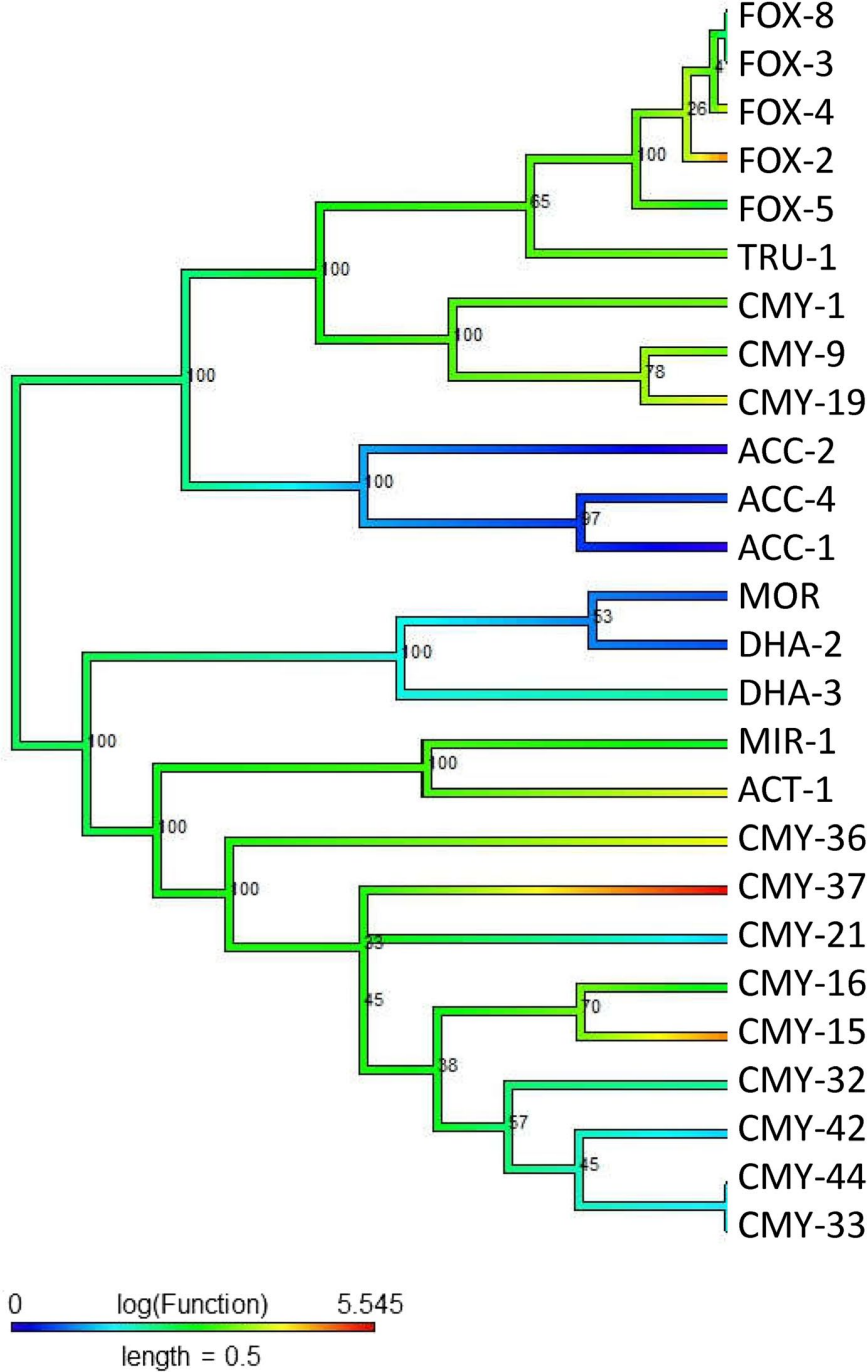
Class C (Cefoxitin): The midpoint‐rooted phylogenetic tree was constructed by maximum‐likelihood method based on the alignment. Bootstrap values are shown on each node. The phylogenetic tree contains class C β‐lactamases. The color of the branch (also in scale bar) indicates functional activity of enzymes against cefoxitin β‐lactam antibiotic. Blue through red color indicated susceptible to resistance functional activity

We also found evidence of the convergent functional evolution of β‐lactamases of class C. Subject to the cefoxitin antibiotics (Figure 4), ancestral function was estimated as moderate resistance (green color) to cefoxitin, but the distantly related enzymes, such as FOX‐2, CMY‐37, and CMY‐15, show evidence of convergent evolved resistance. Additionally, we found evidence that the enzymes ACC‐1, ACC‐2, ACC‐4, MOR, and DHA‐2 independently evolved increased sensitivity to cefoxitin (decreasing resistance).

Piperacillin‐TZB is a combination of piperacillin antibiotics and tazobactam inhibitors. The evolutionary connection among the new enzymes and their functional growth can be observed in Figure S3. This analysis has been conducted on 16 β‐lactamase enzymes that contained ancient resistant functional characteristics of piperacillin‐TZB. The modern enzymatic function of CMY‐9, DHA‐2, ACT‐1, and CMY‐32 has susceptible characteristics, and our analysis suggests that these enzymes have independently developed this susceptibility. As compared to ancestral function, the modern enzymes FOX‐5, CMY‐19, ACC‐4, ACC‐1, and CMY‐36 are strongly resistant to piperacillin‐TZB, and this again has evolved through a convergent evolution.

#### 3.1.4 Class D

**Figure 5:**
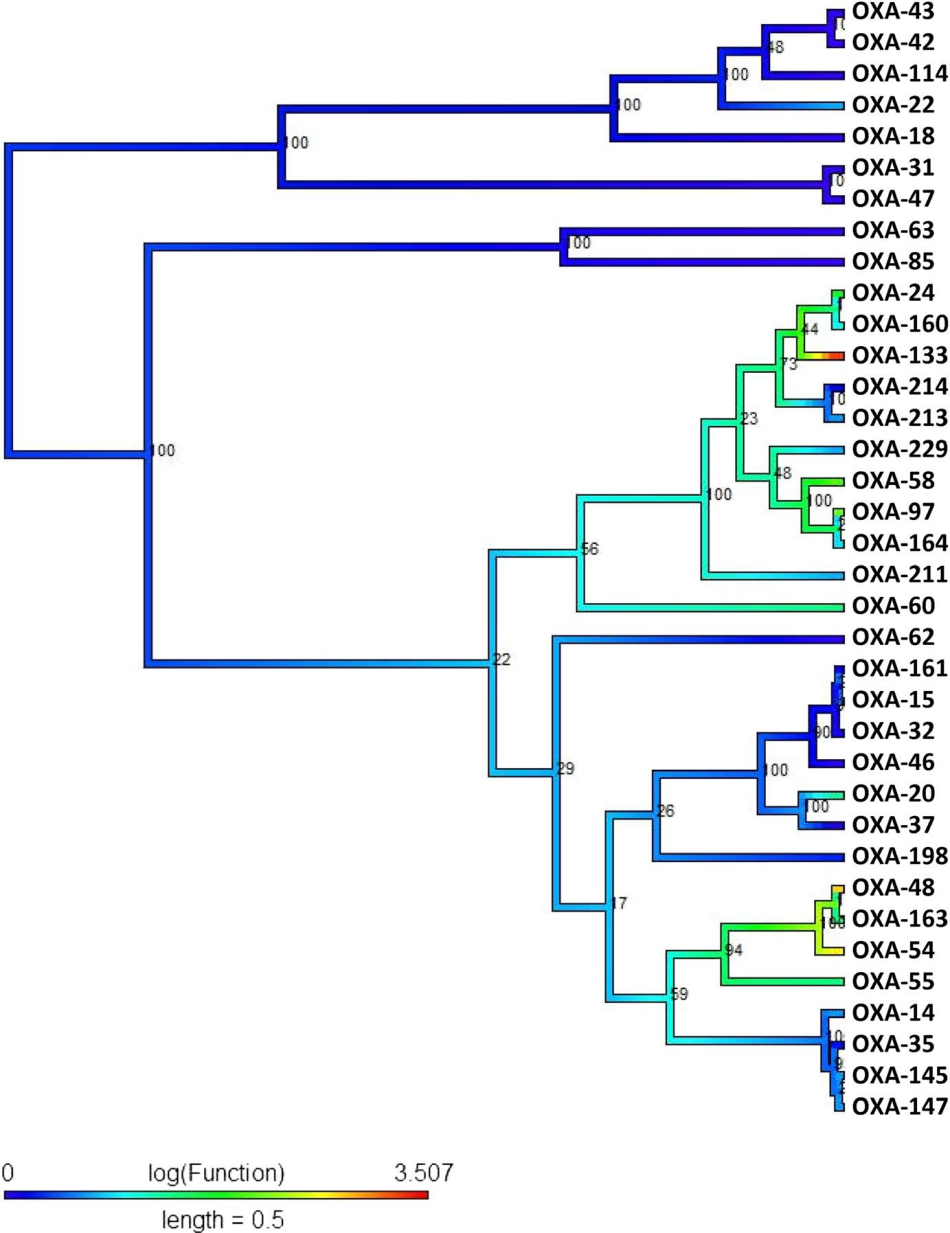
Class D (Imipenem): The midpoint‐rooted phylogenetic tree was constructed by maximum‐likelihood method based on the alignment. Bootstrap values are shown on each node. The phylogenetic tree contains class D β‐lactamases. The color of the branch (also in scale bar) indicates functional activity of enzymes against imipenem β‐lactam antibiotic. Blue through red color indicated susceptible to resistance functional activity. *Blue* indicates susceptible, *Green* moderately resistant, and *red* highly resistant

The antibiotic‐resistant function of the oxacillin molecular class D, with respect to imipenem, has evolved convergently in at least two lineages (Figure 5). The inferred ancestral state is that the functional activity of ancestral enzymes was highly susceptible to carbapenem (Imipenem) which remains an attribute of the vast majority of forms of the enzyme. However, some enzymes (OXA‐24, OXA‐160, OXA‐133, OXA‐58, OXA‐60, OXA‐20, OXA‐48, OXA‐163, OXA‐54, and OXA‐55) have lost their ancestral functional characteristics and evolved resistant to imipenem antibiotics. In particular, the enzyme OXA‐133 is highly resistant (red color) to imipenem, and since this enzyme is deeply nested in a clade which shows slightly increased levels of resistance over the ancestral state, this may suggest that antibiotic resistance in OXA‐133 has evolved in a “rachet‐like” manner as moderate resistance sets the stage for high levels of resistance in later descendants. Such a “stepped” evolution of toxin resistance (of which antibiotic resistance is type) has previously been demonstrated in the evolution of resistance to tetrodotoxin in salamanders (Hanifin & Gilly, 2015), and the generality of such a mechanism is worth further investigation. The imipenem‐resistant class D enzymes appear to have evolved on three separate occasions, and in combination with the other results discussed above, this highlights the remarkable frequency of convergent evolution in β‐lactamase‐mediated antibiotic resistance.

### 3.2 Convergent amino acid residue identification

We investigated whether the convergent functional evolution we observed at the intraclass level of β‐lactamase superfamilies was mediated by underlying convergence at the molecular sequence level (i.e., whether the same mutations conferred resistance). We attempted to identify amino acid residues that may be responsible for functional changes using a python script (provided in Appendix S1) which looks for convergent mutations that co‐occur on the phylogeny with each similar change in the functional activity. However, we did not identify any strong candidate amino acid residue. This may be due to resistance being conferred by changes in (a) the 3D structure of the enzymes (Molina‐Mora & Morgan, 2016), (b) combinatorial effects of multiple amino acid changes, but a different combination in each case, (c) properties that are shared among multiple different amino acids, for instance, if changes in charge were important regardless of the specific amino acid that caused the change, or (d) homologous sequence identity/similarity threshold criteria. Therefore, future work to identify the molecular changes responsible for modifying the functional activity of these enzymes will require a more targeted approach that takes full account of the range of molecular mechanisms of protein evolution. We are therefore not able to report the underlying molecular basis of the functional convergence we document, but we can affirm that neither the change of a single amino acid nor a fixed combination of changes is responsible for all the functional changes we document here. This has crucial implications for the identification of resistant phenotypes, as it suggests that it may not be possible to develop sequence‐based bioinformatic approaches to identifying resistance, which will therefore require more resource‐intensive (but accurate) methods such as functional assays.

## 4 CONCLUSION

In this study, we present evidence of frequent convergent functional evolution of β‐lactamase function conferring resistance to a broad range of commonly used antibiotics. Functional variation and convergent similarities occur in distant related enzymes, indicating that resistance to antibiotics mediated by β‐lactamase cannot necessarily be inferred by sequence similarity alone or by the phylogenetic relationship. We therefore suggest that, although bioinformatic tools to extract antibiotic resistance information from amino acid sequences are in principle more efficient than laboratory functional tests, they are ineffective and functional assays are essential to assess antibiotic resistance. Functional convergent evolution appears to be a common pattern of antibiotic resistance (at least for β‐lactamase) and may therefore provide a useful comparative system for studies of convergence across different levels of life. In addition, testing three‐dimensional molecular structures for structural similarity and analyzing biological pathways that reflect the functional relationship could open the way to other possibilities for predicting antibiotic resistance. Nonetheless, β‐lactamase evolution demonstrates that the dynamics of convergent evolution can differ across different levels of life (e.g., from primary sequence structure to function) in a medically important system, providing opportunities to apply our evolutionary understanding to the management of infections.

## CONFLICT OF INTEREST

None declared.

## Data Availability

This article does not contain data.
