# Supplementary material for: The functional convergence of antibiotic resistance in β‐lactamases is not conferred by a simple convergent substitution of amino acid
Source: Evol Appl. 2019 Jul 18;12(9):1812–22. doi: 10.1111/eva.12835 (PMC6752183; doi:10.1111/eva.12835)
Supplement: Supplementary file 1 [file EVA-12-1812-s001.docx]

**Supplementary File**


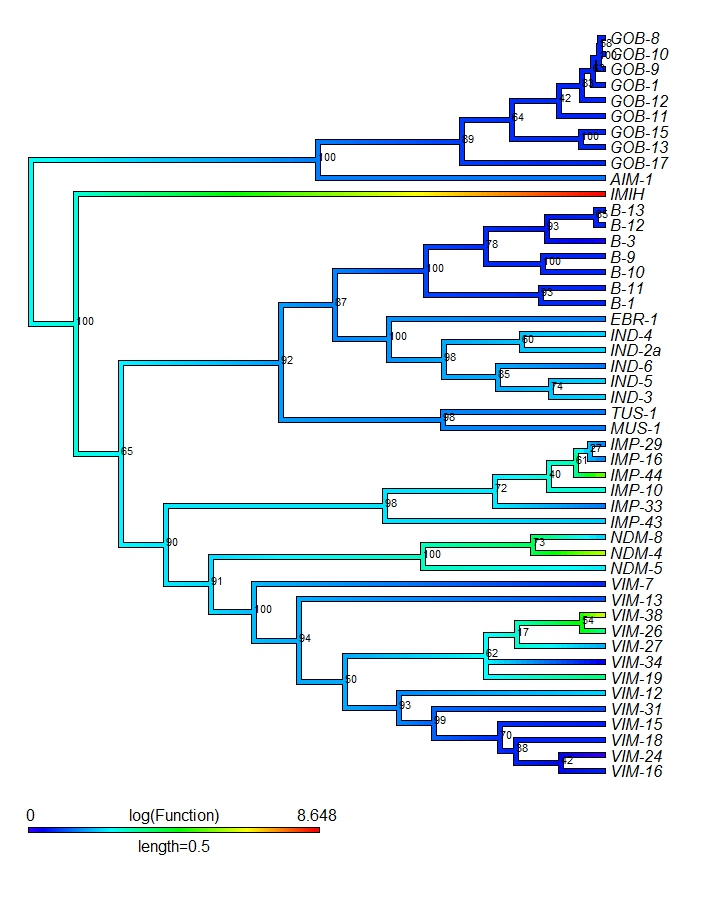


**Fig. S1: Class B (Imipenem):** The midpoint rooted phylogenetic tree was constructed by maximum-likelihood method based on the alignment. Bootstrap values are shown on each node. The phylogenetic tree contains class B β-lactamases. The color of the branch (also in scale bar) indicated functional activity of enzymes against Imipenem β-lactam antibiotic. Color scheme and annotations are as in Fig .1.


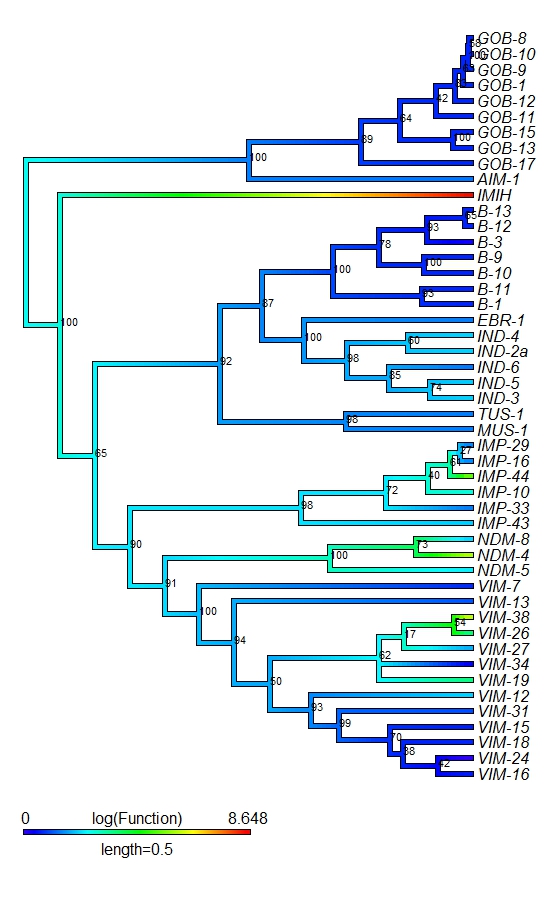


**Fig. S2: Class B (Meropenem):** The midpoint rooted phylogenetic tree was constructed by maximum-likelihood method based on the alignment. Bootstrap values are shown on each node. The phylogenetic tree contains class B β-lactamases. The color of the branch (also in scale bar) indicated functional activity of enzymes against Meropenem β-lactam antibiotic. Color scheme and annotations are as in Fig .1.


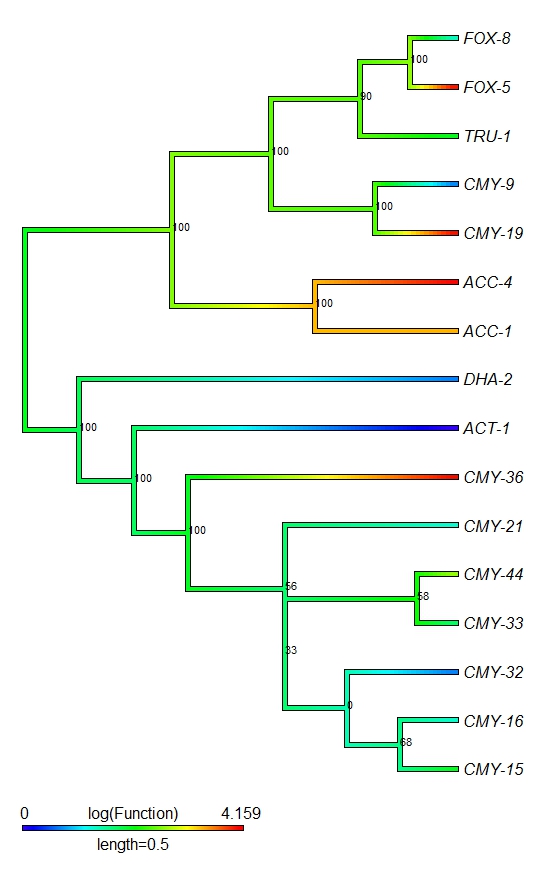


**Fig. S3: Class C (Piperacillin-TZB):** The midpoint rooted phylogenetic tree was constructed by maximum-likelihood method based on the alignment. Bootstrap values are shown on each node. The phylogenetic tree contains class C β-lactamases. The color of the branch (also in scale bar) indicates the functional activity of enzymes against Piperacillin-TZB β-lactam antibiotic. Blue through red color indicated susceptible to resistance functional activity. *Blue* indicates susceptible, *Green* moderate resistant and *red* highly resistant.


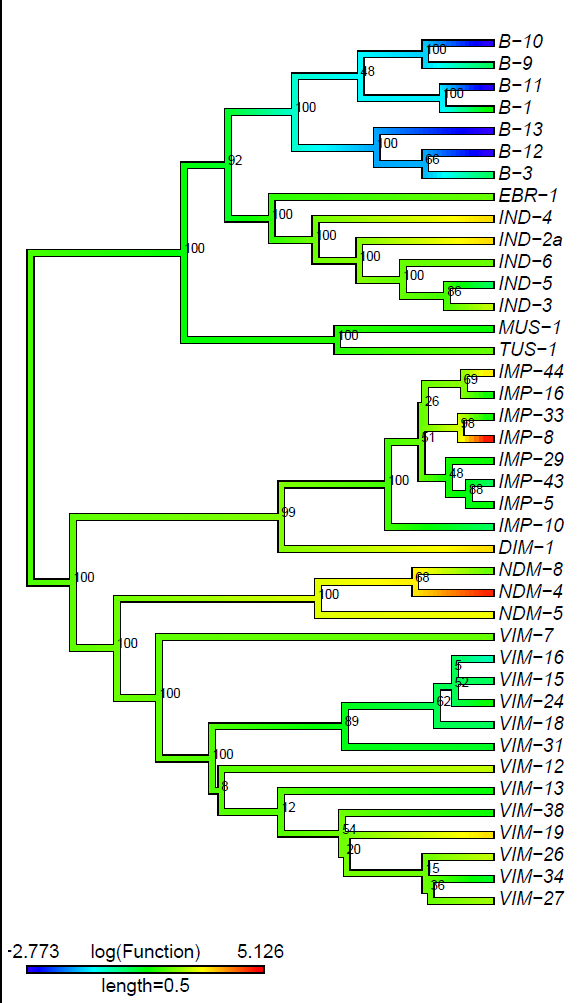


**Fig. S4: Class B (B1, Imipenem):** The midpoint rooted phylogenetic tree was constructed by maximum-likelihood method based on the alignment. Bootstrap values are shown on each node. The phylogenetic tree contains sub-group B1 β-lactamases. The color of the branch (also in scale bar) indicates the functional activity of enzymes against Imipenem β-lactam antibiotic. Blue through red color indicated susceptible to resistance functional activity. *Blue* indicates susceptible, *Green* moderate resistant and *red* highly resistant.


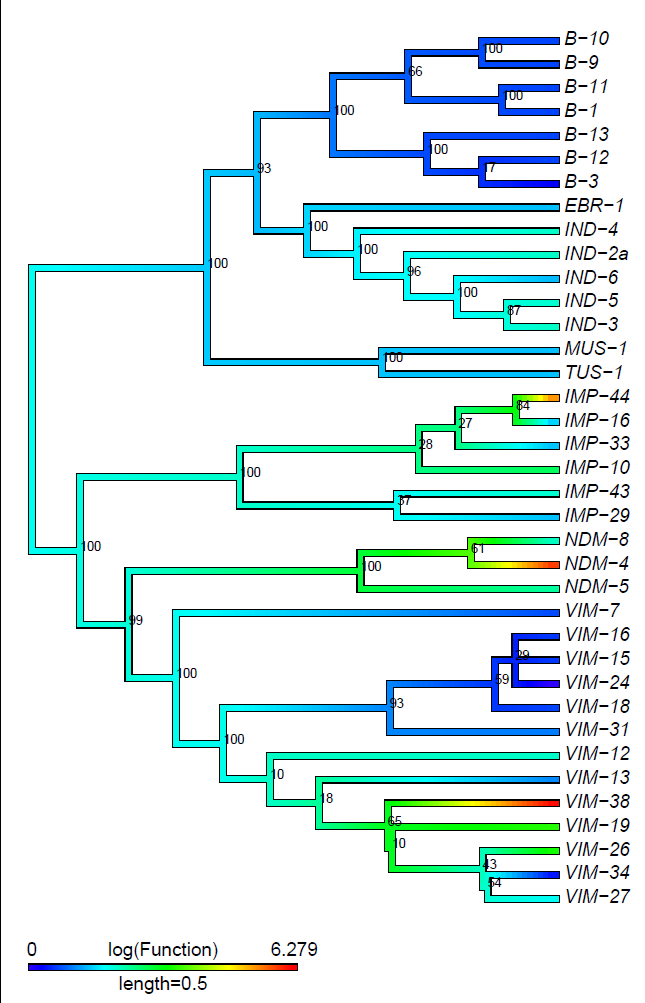


**Fig. S5: Class B (B1, Meropenem): `**The midpoint rooted phylogenetic tree was constructed by maximum-likelihood method based on the alignment. Bootstrap values are shown on each node. The phylogenetic tree contains sub-group B1 β-lactamases. The color of the branch (also in scale bar) indicates the functional activity of enzymes against Meropenem β-lactam antibiotic. Blue through red color indicates susceptible to resistance functional activity. *Blue* indicates susceptible, *Green* moderate resistant and *red* highly resistant.


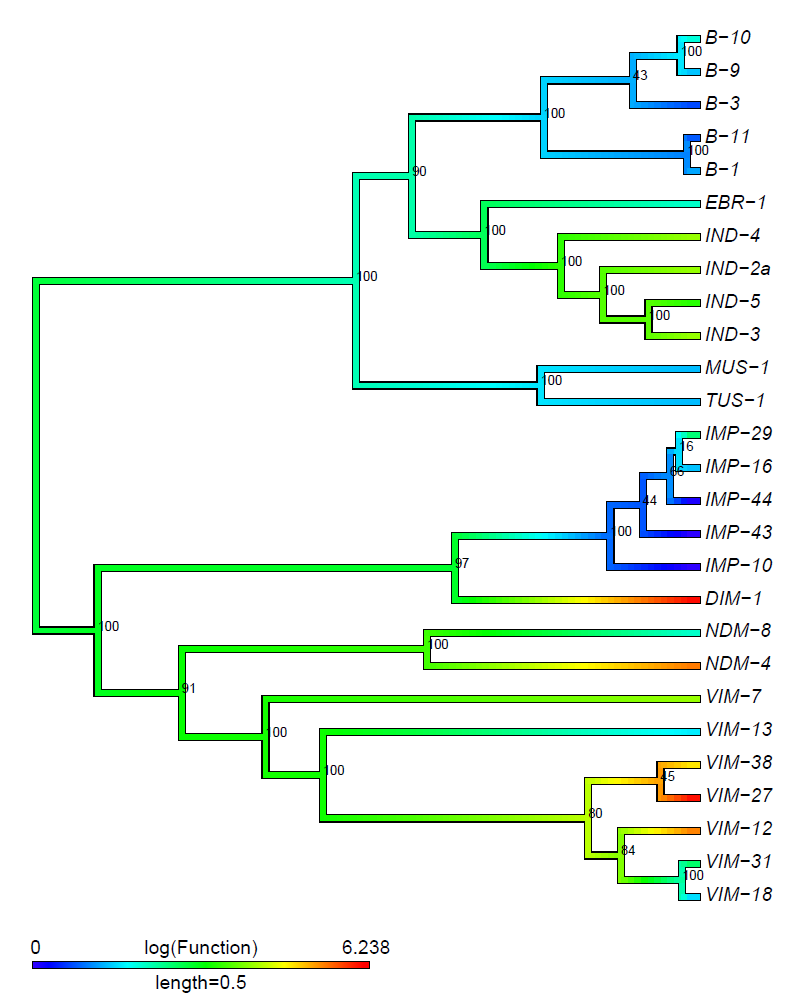


**Fig. S6: Class B (B1, Piperacillin): `**The midpoint rooted phylogenetic tree was constructed by maximum-likelihood method based on the alignment. Bootstrap values are shown on each node. The phylogenetic tree contains sub-group B1 β-lactamases. The color of the branch (also in scale bar) indicated functional activity of enzymes against Piperacillin β-lactam antibiotic. Blue through red color indicated susceptible to resistance functional activity. *Blue* indicates susceptible, *Green* moderate resistant and *red* highly resistant.


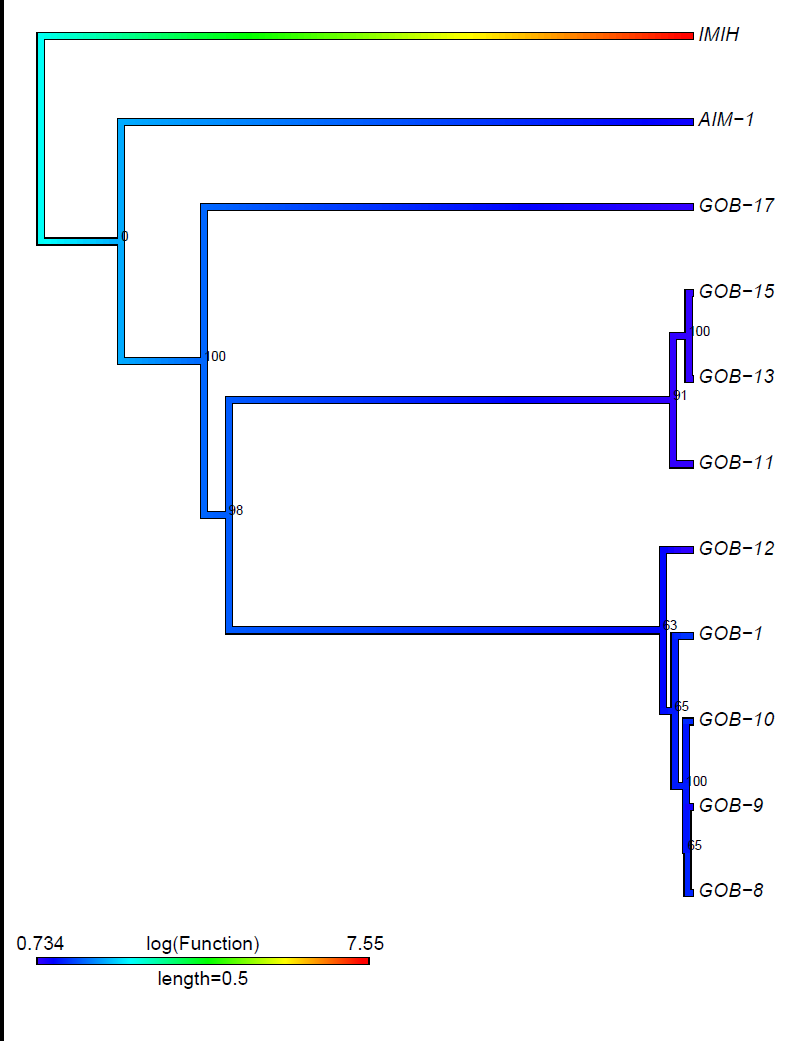


**Fig. S7: Class B (B3, Imipenem):** The midpoint rooted phylogenetic tree was constructed by maximum-likelihood method based on the alignment. Bootstrap values are shown on each node. The phylogenetic tree contains sub-group B3 β-lactamases. The color of the branch (also in scale bar) indicated functional activity of enzymes against Imipenem β-lactam antibiotic. Blue through red color indicated susceptible to resistance functional activity. *Blue* indicates susceptible, *Green* moderate resistant and *red* highly resistant.


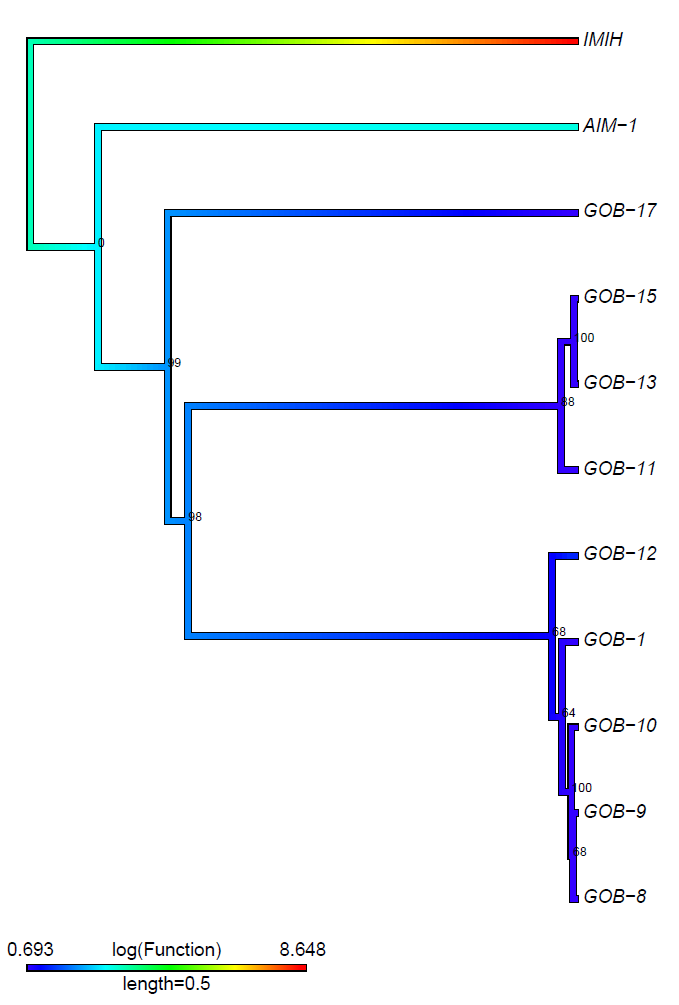


**Fig. S8: Class B (B3, Meropenem):** The midpoint rooted phylogenetic tree was constructed by maximum-likelihood method based on the alignment. Bootstrap values are shown on each node. The phylogenetic tree contains sub-group B3 β-lactamases. The color of the branch (also in scale bar) indicated functional activity of enzymes against Meropenem β-lactam antibiotic. Blue through red color indicated susceptible to resistance functional activity. *Blue* indicates susceptible, g*reen* moderate resistant and *red* highly resistant.


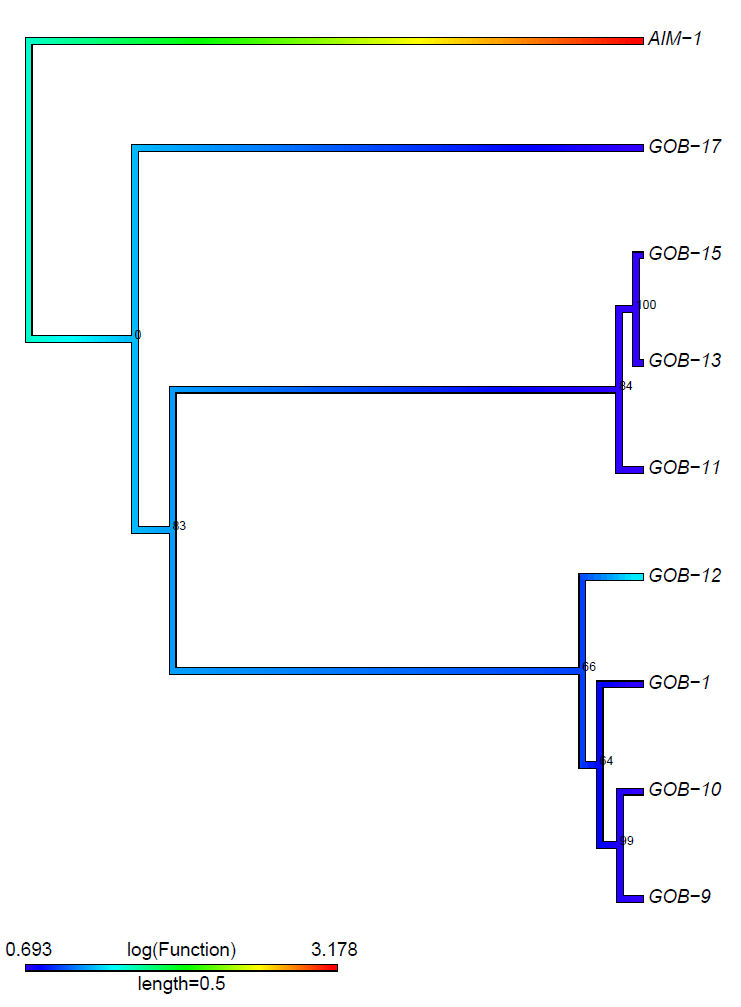


**Fig. S9: Class B (B3, Piperacillin):** The midpoint rooted phylogenetic tree was constructed by maximum-likelihood method based on the alignment. Bootstrap values are shown on each node. The phylogenetic tree contains sub-group B3 β-lactamases. The color of the branch (also in scale bar) indicated functional activity of enzymes against Piperacillin β-lactam antibiotic. Blue through red color indicated susceptible to resistance functional activity. *Blue* indicates susceptible, g*reen* moderate resistant and *red* highly resistant.


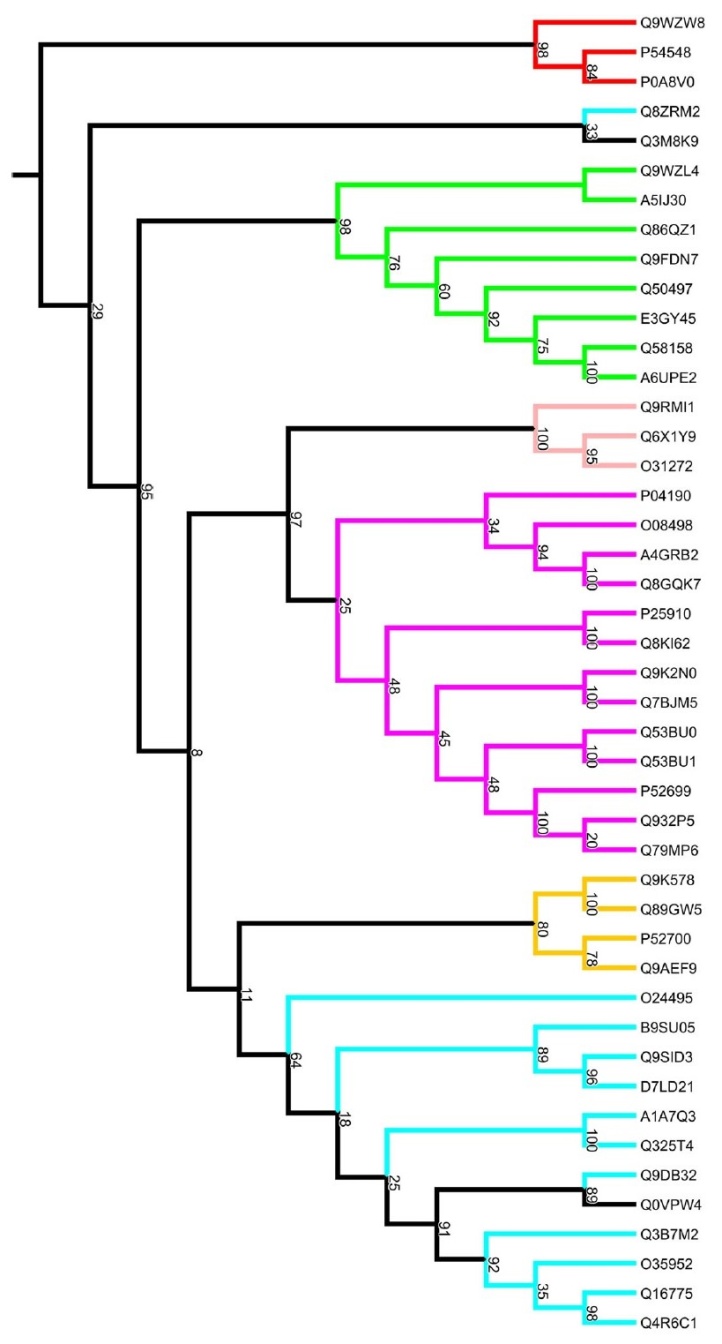


**Fig. S10: Phylogenetic tree of class B β-lactamases** (11). Enzymes groups are colour coded by function as follows- Magenta, Pale pink and orange color indicates subclass B1, B2 and B3 metallo-β-lactamases respectively. *Red* ribonucleases, *cyan* glyoxalase IIs, *green* A-type flavoproteins and *black* no functions assigned.

**Python script to identify the convergent amino acid residue**

#!/usr/bin/python

import re

import sys

def checkArguments():

"""

Function to check arguments numbers, if it's diffent than 4, print help message and exit

"""

if len(sys.argv) -1 != 4:

sys.stderr.write("differentAA.py [Input :paml rst file] [Input : convergent species list comma separated (without blank space)] [Input :target list comma separated (without blank space)] [output fileName]\n")

sys.stderr.write("Detect Amino Acid present in convergent species and not in the orther\n")

sys.stderr.write("Input:\n")

sys.stderr.write("------\n")

sys.stderr.write("1 - Paml rst file : result file from Paml\n")

sys.stderr.write("2 - List of the convergent species. The list must be comma separated witout blank space : 9606,8292, ...\n")

sys.stderr.write("3 - List of the species, not specificly convergent, but where you want to look, if the species present the same Amino Acid. The list must be comma separated witout blank space : 9606,8292, ...\n")

sys.stderr.write("Output:\n")

sys.stderr.write("------\n")

sys.stderr.write(" 3 Files :\n")

sys.stderr.write(" [output file name].convergent.txt : Contains common positions betweens convergent species \n")

sys.stderr.write(" [output file name].common.txt : Contains common positions betweens commons species (see input)\n")

sys.stderr.write(" [output file name].logs.txt : different kind of log from the script, to understand how it create the different files \n")

sys.stderr.write("olivier.chabrol@univ-amu.fr\n")

sys.exit(1);

def readPamlFile(pamlFile, convergentList, targetList, outFileName):

"""

Function to read Paml formated file, generate 3 files

"""

fastas = {}

try:

fichierPaml = open(pamlFile, "r")

fichierSortie = open(outFileName + ".convergent.txt", "w")

fichierSortieCommon = open(outFileName + ".common.txt", "w")

log = open(outFileName + ".logs.txt", "w")

startReadTree = 0

startReadSeq = 0

seqLineNumber = 0

tree = ""

for line in fichierPaml:

line = line[:-1]

# detection de l'arbre

if line.find("TreeView") != -1:

startReadTree = 1

else:

if startReadTree == 1 and line == "":

startReadTree = 0

if startReadTree == 1:

line = re.sub("\) ", ")", line)

line = re.sub(" \)", ")", line)

line = re.sub(" ,", ",", line)

# PAML rajoute le numero du noeud _ le nom de la sequence par ex:

# 1_HOMO, il faut supprimer ce [0-9]*_

line = re.sub("[0-9]+_", "", line)

tree = line

if line.find("List of extant and reconstructed sequences") != -1:

startReadSeq = 1

else:

if startReadSeq == 1:

seqLineNumber += 1

if line.find("Overall") != -1:

startReadSeq = 0

elif line != "" and seqLineNumber > 3:

line = re.sub(" ", "*", line)

line = re.sub(" ", "", line)

line = re.sub("[\*]+", " ", line)

fastaHeader = line[0:line.find(" ")]

# PAML reindex the nodes of the tree for him, but put a string node#[0-9]* before ne name of the node in the re construct ancestral request

# we need to delete it

if fastaHeader.find("node#") == 0:

fastaHeader = fastaHeader[5:]

fasta = line[line.find(" ") + 1:]

if fastaHeader != "":

fastas[fastaHeader] = fasta;

except IOError as e:

print ("I/O error({0}): {1}".format(e.errno, e.strerror))

# find the same character in the convergent genes

firstGeneFasta = fastas[convergentList[0]]

fastaSize = len(firstGeneFasta)

difConv = False

difOther = False

dif = False

for i in range(0, fastaSize):

char = firstGeneFasta[i]

difConv = False

difOther = False

dif = False

for k in fastas:

if k != convergentList[0]:

# if the gene is convergent

if k in convergentList:

if fastas[k][i] != char:

difConv = True

log.write("At position " + str(i) + " the Amino Acid is different\n")

break

elif k in targetList:

if fastas[k][i] == char:

difOther = True

log.write("At position " + str(i) + " the Amino Acid of gene " + k + " is the same as the first convergent one (" +convergentList[0] + ")\n")

break

if difConv or difOther:

fichierSortie.write("*")

else:

fichierSortie.write(char)

for g in range(1, len(convergentList)):

if fastas[convergentList[g]][i] != char:

dif = True

if dif:

fichierSortieCommon.write("*")

else:

fichierSortieCommon.write(char)

fichierPaml.close()

fichierSortie.close()

fichierSortieCommon.close()

log.close()

print (outFileName + ".convergent.txt generated")

print (outFileName + ".common.txt generated")

print (outFileName + ".log.txt generated")

if __name__ == '__main__':

checkArguments()

readPamlFile(sys.argv[1], sys.argv[2].split(","), sys.argv[3].split(","), sys.argv[4])
